# Liquid Metal‐Based Multifunctional Micropipette for 4D Single Cell Manipulation

**DOI:** 10.1002/advs.201700711

**Published:** 2018-05-03

**Authors:** Yu Ting Chow, Tianxing Man, Giovanny F. Acosta‐Vélez, Xiongfeng Zhu, Ximiao Wen, Pei‐Shan Chung, Tingyi “Leo” Liu, Benjamin M. Wu, Pei‐Yu Chiou

**Affiliations:** ^1^ Mechanical and Aerospace Engineering Department University of California Los Angeles CA 90095 USA; ^2^ Bioengineering Department University of California Los Angeles CA 90095 USA; ^3^ School of Dentistry University of California Los Angeles CA 90095 USA

**Keywords:** cell manipulation, dielectrophoresis, electrorotation, liquid metals

## Abstract

A novel manufacturing approach to fabricate liquid metal‐based, multifunctional microcapillary pipettes able to provide electrodes with high electrical conductivity for high‐frequency electrical stimulation and measurement is proposed. 4D single cell manipulation is realized by applying multifrequency, multiamplitude, and multiphase electrical signals to the microelectrodes near the pipette tip to create 3D dielectrophoretic trap and 1D electrorotation, simultaneously. Functions such as single cell trapping, patterning, transfer, and rotation are accomplished. Cell viability and multiday proliferation characterization has confirmed the biocompatibility of this approach. This is a simple, low‐cost, and fast fabrication process that requires no cleanroom and photolithography step to manufacture 3D microelectrodes and microchannels for easy access to a wide user base for broad applications.

## 1 Introduction

Rapid progresses in micro‐and nanomanufacturing have resulted in the advancements in numerous research and application fields in the past few decades. High‐resolution patterning capability and low‐cost mass production have revolutionized the fields of integrated circuits and microelectromechanical systems (MEMS). The invention of soft lithography further extended the impact in the fields of microfluidics and BioMEMS.^1^, ^2^, ^3^, ^4^ However, conventional micro‐ and nanomanufacturing methods have several fundamental limitations. First, they are mainly 2D patterning techniques. 3D structures need to be created through layer‐by‐layer deposition with limited control in the third dimension. Second, they are planar techniques that cannot pattern features on nonplanar surfaces.

Glass micropipette pulling techniques, despite the rapid progresses of micro‐ and nanofabrication, have not been replaced by modern microfabrication and are still widely used in various fields, especially in biology.^5^, ^6^, ^7^ Micropipette fabrication process is simple, rapid, and low cost. It requires only a capillary tube and a single pulling step. The diameter of the tip and opening can be continuously tuned from micrometers down to nanometers by changing the pulling parameters. Microcapillary pipettes are ideal for single cell manipulation, such as cell picking and loading. Submicron‐sized capillary tubes allow microinjection and delivery of exogeneous materials into cells by piecing through cell membranes.^8^, ^9^, ^10^, ^11^, ^12^ The long capillary filled with aqueous liquid can also function as an electrode for measuring cell properties or performing electroporation.^13^, ^14^ Moreover, the functionality of microcapillary could be increased by pulling a multibarrel capillary to provide independent control of different fluid flows for microfluidic applications, such as the creation of droplets with complex content.^15^ Multifunctional micropipettes can also be accomplished by jointly pulling glass capillaries with other materials such as carbon fibers, or metal nanoparticles.^16^ However, the electrical properties, such as the electrical conductivity of the pulled wires, are not easy to control, and the postprocessing of the composite pipettes before they can be used could be complicated.^17^, ^18^, ^19^, ^20^

Here, we demonstrated a liquid metal‐filled multifunctional microcapillary pipette that is easy to fabricate and can provide electrodes with high electrical conductivity for high‐frequency electrical measurement and excitation. To demonstrate its potential applications, we show that this pipette can provide 4D single cell manipulation by applying multifrequency (up to 10 MHz), multiphase electrical signals across a long pipette (few centimeters) to create dynamic electric field patterns near the pipette tip.

## 2 Results

### 2.1 Fabrication of Liquid Metal‐Filled Microcapillary Pipette

**Figure 1:**
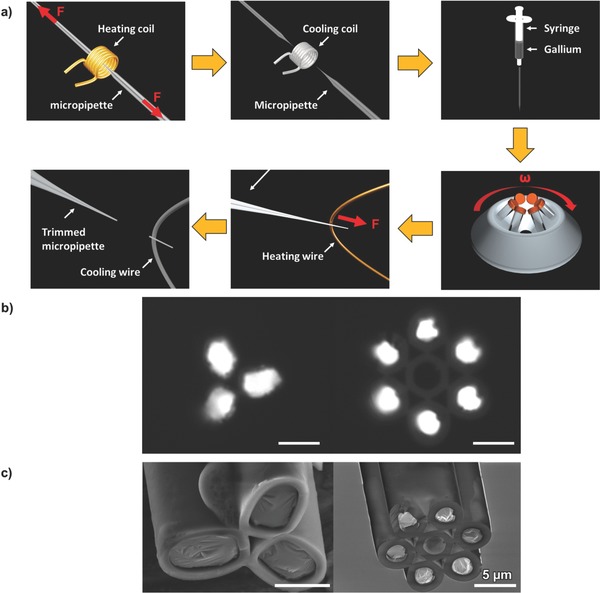
Fabrication process of liquid metal‐filled multifunctional micropipette. a) A multibarrel glass capillary is heated and pulled with a custom‐made micropipette puller. The micropipette is then filled with liquid gallium using a syringe and centrifuged to drive the gallium to the tip. After that, the micropipette is trimmed by a microforge to have a desired tip diameter. b) Microscopic images of the pulled multibarrel pipettes selectively filled with liquid metal in desired channels. The center channel of the 7‐barrel pipette is kept empty to allow fluid delivery. c) Scanning electron microscopic images of the pipettes. Note that the surface of liquid metal exposed to air is quickly oxidized to form a thin solid film (≈1 nm thick), which helps stabilize the shape.^21^ Scale bars in all figures: 5 µm.

The fabrication approach of the proposed liquid metal‐filled, multibarrel glass capillary is shown in **Figure** **1**a. The processes require only capillary pulling, selective filling of liquid gallium into the pulled micropipette to create microelectrodes, and trimming the micropipette to have a desired diameter. In contrast to conventional microfabrication, this approach does not require any cleanroom and photolithography processes to create these long, patterned, and 3D microelectrodes. This significantly saves the manufacturing cost, speeds up the design process, and allows rapid adjustment of electrode size. As a demonstration of this new fabrication method, 3‐ and 7‐barrel micropipettes are fabricated as shown in Figure 1b,c. The measured electrical resistance of a cm‐long, tapered liquid metal electrode with a 7 µm opening tip is 12 to 15 Ω. This low electrical resistance of the fabricated electrode permits high‐frequency electrical excitation and measurement near the micropipette tip without losing power in the long electrical conduction wire. In this paper, a 3‐barrel micropipette is further used to perform 4D single cell manipulation by simultaneously applying a 10 MHz electrical signal to create a 3D dielectrophoretic cell trap, and a 400 kHz signal to induce electrorotation (EOT) on the trapped cell.

### 2.2 4D Single Cell Manipulation

#### 2.2.1 Working Principle of Dielectrophoresis (DEP)

DEP is a phenomenon in which a particle in a nonuniform electric field is polarized and interacts with the applied field to generate a net force moving it toward a strong electric field region if it is more polarizable than the medium (positive DEP), or to a weak field region if the particle is less polarizable than the medium (negative DEP). The time‐averaged DEP force on a particle in a nonuniform electric field (E‐field) is given by the following equation^22^

(1) $$\left\langle F_{DEP}\right\rangle =2\pi r^{3}\;\varepsilon_{m}Re\left(f_{cm}\right)\nabla E^{2}$$

where ***r*** is the radius of the particle, ***ε***_**m**_ is the permittivity of the medium, *E* is the E‐field, and **Re(** ***f***_**cm**_ **)** is the real part of the Clausius–Mossotti (CM) factor. The CM factor, ***f***_**cm**_ **(** ***ω*** **)** is

(2) $$f_{cm}\;(\omega )=\frac{\varepsilon_{p}^{*}-\varepsilon_{m}^{*}}{\varepsilon_{p}^{*}+2\varepsilon_{m}^{*}},\;\varepsilon_{p}^{*}=\varepsilon_{p}-j\frac{\sigma_{p}}{\omega }\;\mathrm{and}\;\varepsilon_{m}^{*}=\varepsilon_{m}-j\frac{\sigma_{m}}{\omega }$$

where $\varepsilon_{p}^{*}$ and $\varepsilon_{m}^{*}$ are the complex permittivity of the particle and medium, respectively, ***j*** is $\sqrt{-1}$, ***σ***_**p**_, and ***σ***_**m**_ is the conductivity of the particle and medium, respectively, and ***ω*** is the angular frequency of the applied E‐field. The magnitude of DEP force depends on the gradient, the frequency, the amplitude of the E‐field, and the size of the particle. DEP has been widely used for cell manipulation in the past decade for cell trapping and sorting applications. DEP manipulation of biological cells in aqueous media is typically carried out with electrical signals in the frequency range between 100 kHz and 10 MHz. Within this frequency range, cell viability can be maintained, and different types of cells show significant differences in their dielectric properties and DEP responses.

**Figure 2:**
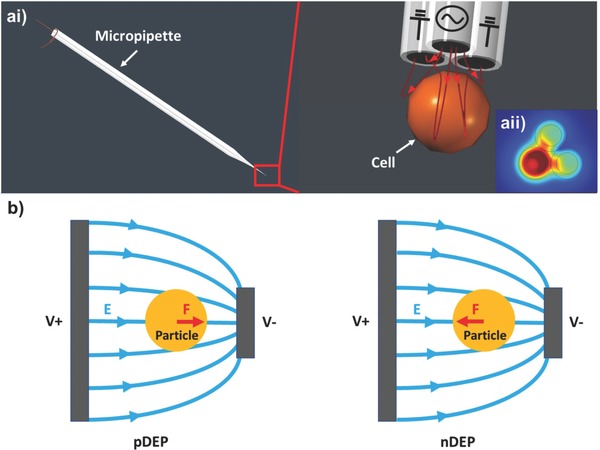
The schematic of using the liquid metal electrode pipette for DEP trap. a) A 3‐barrel glass capillary is filled with gallium, and copper wire is inserted into each barrel for the electrical connection to the function generator. DEP force is generated by applying AC signal to the three electrodes. The insert shows the electric field distribution near the pipette tip. b) Positive DEP attracts particle toward the strong E‐field region, while negative DEP repels particle away from the strong E‐field region.

The schematic of a 3‐barrel pipette used for DEP demonstration is shown in **Figure** **2**a. Gallium is filled in all three channels to form electrodes. AC electrical signals are applied to these three gallium electrodes to create a nonuniform electric field distribution near the tip to induce DEP forces on nearby objects. If a positive DEP force is induced on a cell nearby, it will be attracted to the strong electric field region at the center of the pipette tip.

#### 2.2.2 Working Principle of Electrorotation (ROT)

ROT is a phenomenon in which a particle rotates in a rotating electric field. It is the result of the phase delay between the induced electric dipole on the particle and the external rotating field. This delay creates an angle between the directions of the induced dipole and the external field, which results in the generation of a constant mechanical torque rotating the particle. What determines the direction and the magnitude of the torque on a particle are the electric field strength, frequency, and the dielectric composition of the particles.

DEP and ROT have similar physical origins, both based on the interactions between the field‐induced dipole on a particle and their relationship with the external field. Based on cells' dielectric signatures, both DEP and ROT have been shown to be able to provide label‐free characterization of different cell types^23^, ^24^ or cells at different growth phases.^25^

**Figure 3:**
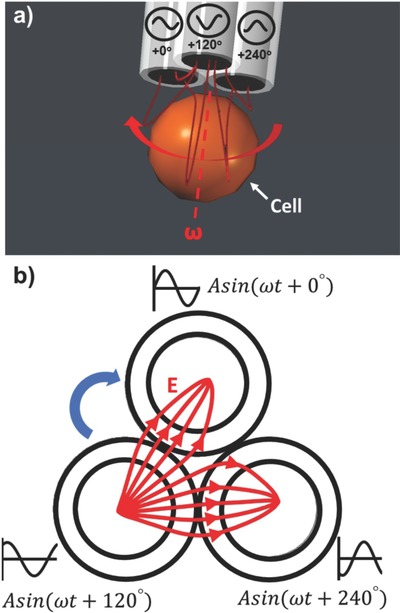
Schematic of electrorotation near the pipette tip. Three AC signals with 0°, 120°, and 240° phase differences are applied to the three electrodes.

The 3‐barrel multifunctional pipette was also utilized to rotate the trapped cell in the axial direction by applying 120° phase difference AC signals to the three electrodes (**Figure** **3**). The electrorotation torque **Γ** is given by the following equation^22^

(3) $$\left\langle \Gamma \right\rangle =-4\pi r^{3}\;\varepsilon_{m}Im\left(f_{cm}\right){\left|E\right|}^{2}$$

where **Im(** ***f***_**cm**_ **)** is the imaginary part of the CM factor.

#### 2.2.3 DEP Force Calibration

**Figure 4:**
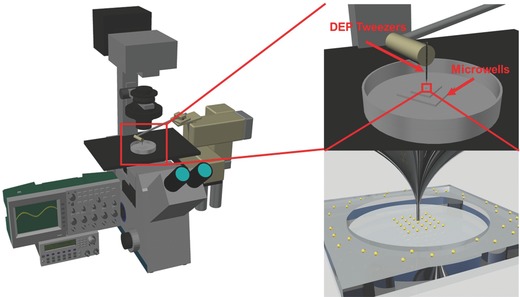
System setup. The system consists of a micromanipulator, an inverted microscope, a multifunctional pipette, an oscilloscope, and a function generator. The micropipette is mounted on the micromanipulator.

**Figure 5:**
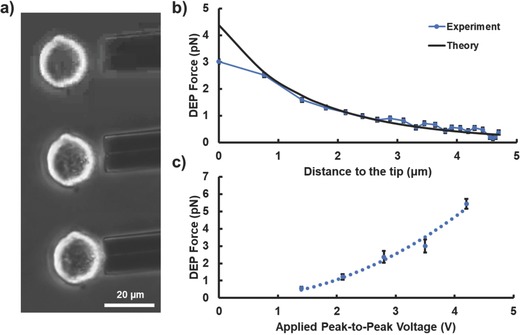
DEP force calibration. a) Snapshots of images captured during a Hela cell trapping process. The DEP force was determined by measuring the velocity of the cell before it touched the tip. a,b) A 10 MHz, 2.8 V_pp_ AC signal is applied. The theory curve is based on the electric field strength simulated by COMSOL Multiphysics near the pipette tip without cell. c) A plot of the maximum DEP force against the applied peak‐to‐peak voltage. These forces are measured right before the cell touches the tip (0 µm).

The characterization of DEP force is carried out by measuring a cell's moving velocity from the captured video snapshots. In the low Reynolds number microfluidic environment, the Stokes' law can be used to estimate the DEP force based on its relationship with cell moving speed, cell size, and fluid viscosity. For a spherical particle, this relationship is given by the following equation

(4) $$F_{Drag}=6\pi rv\eta$$

where ***η*** is the dynamic viscosity of the medium, ***v*** is the velocity of the particle, and ***r*** is the radius of the particle. The dynamic viscosity of the medium is assumed to be 10^−3^ Pa. In an isotonic buffer (8.5% sucrose, 0.3% dextrose, and conductivity 0.028 S m^−1^), the large difference of the electrical conductivity between cell's cytoplasm (≈0.5 S m^−1^) and the surrounding medium (0.028 S m^−1^) gives a live Hela cell significant positive DEP response at 100 kHz. However, for a dead cell, its cell membrane is permeable to ions. The electrical conductivity difference inside and outside the dead cell is small. Dead cell usually has very weak and negative DEP response due to its small, negative, real‐part of the Clausius–Mossotti factor^26^ (refer to Movies S1 and S2 in the Supporting Information). The schematic of the experimental setup is shown in **Figure** **4**. The force measurement results are shown in **Figure** **5**. From Figure 5c, the DEP force increases with voltage, and is proportional to the square of the applied voltage as predicted by the theory. The effective DEP range near a pipette tip is highly dependent upon the separation distance between electrodes. These electrodes produce an electric field distribution similar to an electrical dipole. At distance far larger than the separation distance of the electrodes, electric field strength decays at a rate ≈***1*** **/** ***r**^3^*. The effective DEP range near the pipette is around the separation distance between the electrodes. Outside this range, electric field decays fast and so does the DEP force. For the 3‐barrel pipette, separation distance between electrodes is ≈5 µm. The measured DEP force in Figure 5b also confirmed this and showed that DEP force beyond 5 µm becomes very small. This effective range is smaller than the size of a regular mammalian cell. Thus, only one cell near the tip will be trapped. The electric field distribution near electrodes cannot be predicted by the electric dipole model and needs to be solved numerically. The theoretical force prediction curve shown in Figure 5b is based on the local electric field distribution simulated using COMSOL Multiphysics. Of note is that, since the cell size is larger than the effective field range, the experimental DEP force measured near the tip will be smaller than the prediction since not the whole cell will be under the strong electric field region used for theoretical calculation. That is a potential reason why there is larger deviation of the experimental DEP force from the theoretical trend near the tip.

#### 2.2.4 Cell Trapping and Rotation

**Figure 6:**
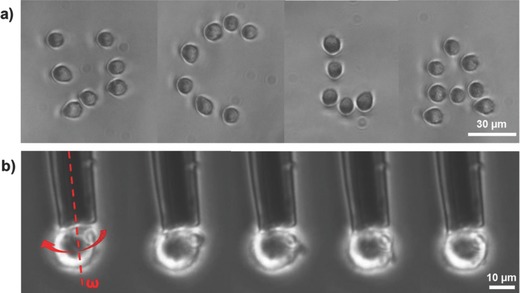
Cell manipulation results. a) A “UCLA” pattern was produced by using the DEP tweezers to trap, transfer, and unload single cells. A 10 MHz, 1.6 V_pp_ AC signal is applied to the tweezers. Ramos B cell was used in this experiment. b) The trapped cell near the tip was rotated by applying AC signals with 0°, 120°, and 240° phase differences, and 3.2 V_pp_ and 400 kHz to the three electrodes (Movie S3, Supporting Information). PANC‐1 cell was used in this experiment. Red dotted line is the cell rotation axis.

**Figure** **6** shows the cell manipulation and electrorotation results. Arbitrary cell patterns can be formed by sequentially trapping, transferring, and releasing single cells as shown in Figure 6a. In Figure 6b, a trapped cell is electrically rotated by superpositioning AC signals that consist of two AC frequencies, two amplitudes, and difference phase delays to the three electrodes on the 3‐barrel pipette (DEP: 10 MHz, 1.6 V_pp_; ROT: 400 kHz, 3.2V_pp_) (Movie S3, Supporting Information). Note that, the experimental setup uses barely a Petri dish as a substrate. All electrodes needed for DEP and ROT manipulation are provided directly by the multifunctional pipette.

#### 2.2.5 Cell Viability Analysis

**Figure 7:**
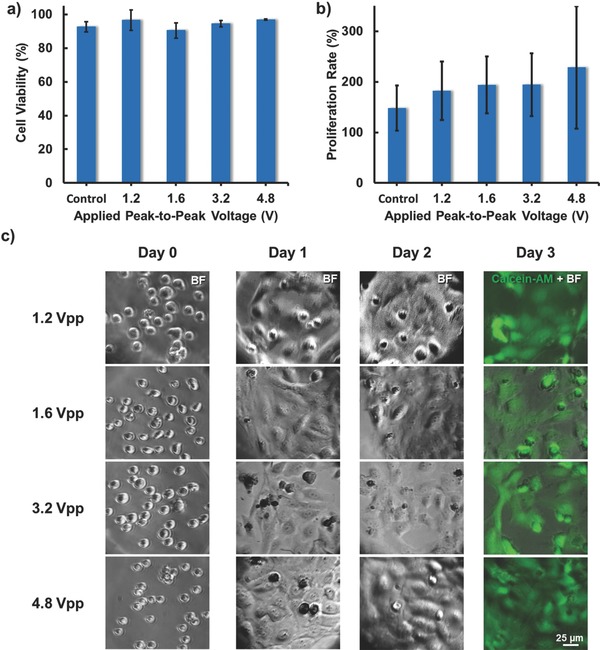
Cell viability results. a) The cell viability test 12 h after the DEP manipulation at different voltages. b) The cell proliferation rate 3 d after the DEP manipulation at different voltages. c) The time lapsed images of cells transferred to microwells at 1.2, 1.6, 3.2, and 4.8 V_pp_, 10 MHz. In day 3, calcein AM was used to identify and count the number of live cell in the microwells. Hela cells are used in this study.

Cell viability test was conducted to investigate the potential impacts on cells after manipulation. The cell viability was evaluated 12 h after the DEP manipulation. The cell viability is defined as the number of calcein AM positive cells over the number of cells in the microwell right after DEP manipulation. In addition, the multiday proliferation rate of the manipulated cells was tracked for 3 d. At day 3, the cells were stained with calcein AM to count the number of live cells. The proliferation rate is defined as the total number of cells (including dead cells) after 3 d over the number of cells in the same microwell right after DEP manipulation. In **Figure** **7**a,b, cell viability is >90% in all cases and the proliferation rate is >180% for all applied voltage. The images in Figure 7c show that the transferred cells could readhere to the substrate and continue to proliferate after manipulation. Note that, the proliferation rate is higher when the applied voltage is higher. Their findings agree with what prior works have suggested that electrical stimulation may induce cell proliferation or even differentiation.^27^, ^28^, ^29^ The proposed multifunctional pipette could have a potential use as a highly localized single cell electrical stimulator for biological studies and other applications, in addition to examples demonstrated in the current work.

## 3 Discussion

The proposed rapid micropipette and microelectrode fabrication approach has high flexibility on fabricating micropipettes of diameters ranging from 1 mm to ≈1 µm. The upper limit is bounded by the diameter of the capillary we used. For the lower limit, a pipette with a diameter as small as 1 µm and gallium filled up to the tip has also achieved.

During our experiment, Gallium remains in liquid state even though the operation temperature is lower than the 30 °C melting point of gallium due to the supercooling effect.^30^ Supercooling is the effect that a liquid does not transit to solid state, even when the temperature is below the freezing point. It has been observed that pure gallium can even stay in liquid state when the temperature is reduced to −28.2 °C.^31^

Electrochemical reactions occur when there is sufficient voltage drop across the electrode and fluid interface capacitance.^32^ Although DEP operation utilizes high‐frequency AC electric field to manipulate cell, there is still a possibility that electrochemical reactions may occur. In our current pipette configuration, the RC charging time is ≈1 µs in an ionic buffer with an electrical conductivity of 0.028 S m^−1^. In our DEP operation conditions at 1.6 V_pp_, 10 MHz frequency, the electrochemical reaction concern should be small. In the electrorotation experiment, we applied a 3.2 V_pp_ and 400 kHz AC signal. There is a possibility that electrochemical reactions occur at the tip, although we did not observe it during our experiment under a microscope. In our experiment, cells after manipulation were transferred to regular physiological buffers. Cell viability and proliferation tests do not show observable toxicity effects resulting from potential electrochemical reactions at the tip. It is expected that electrochemical reactions will have higher chance to occur, same as all other electrokinetics devices, when DEP and ROT operations are conducted at high voltage, in high ionic buffers, or when the micropipette size is further scaled down.

The potential applications of the proposed multifunctional micropipettes are broad. For instance, single cell manipulation has been widely used to characterize the molecular genetics of individual cancer cells,^33^ tumor cell migration,^34^ and characterization of osteogenic differentiation of MC 3T3‐E1 cells.^35^ In addition to providing an effective way for single cell trapping and transport, this tool, in particular, allows users to distinguish different types of cells based on their dielectric signatures (based on DEP or Electrorotation responses) without labeling and selectively pick up cells of desired properties.^36^ This is a small scale cell sorter that can be performed directly on a Petri dish where cells are cultured without any extra devices. This micropipette can also perform cell lysis on site and retrieve the lysed material through the fluidic channel on the multibarrel micropipette for further analysis.

## 4 Conclusion

This work presents a liquid metal‐based multifunctional microcapillary pipette able to provide high‐conductivity electrodes allowing high‐frequency electrical stimulation and measurement. 4D single cell manipulation has been achieved using a multifunctional pipette by supplying multifrequency, multiamplitude, and multiphase electrical signals in the MHz range to create 3D DEP trap and 1D electrorotation, simultaneously. Cell patterning, cell viability analysis, and multiday proliferation characterization have been performed to confirm the single cell manipulation function and biocompatibility of this method. One unique property of this approach is that it does not require any microfabrication and photolithography steps to create microelectrodes, which greatly simplifies the fabrication process, lowers the fabrication cost, and makes this approach easily assessable to wide users, especially in biomedical fields, for broad impacts.

## 5 Experimental Section

*Fabrication of the Liquid Metal‐Based Multifunctional Micropipettes*: The fabrication process is shown in Figure 1. A 3‐ or 7‐ barrel glass capillary (3B100‐75‐10, Sutter Instrument; 7B100F‐6, World Precision Instrument) was pulled with a custom‐built pipette puller. This pulling process was used to produce a small tip of a few microns. Then, the liquid metal electrodes were fabricated by selectively filling gallium with a syringe. In order to ensure the gallium was filled up to the tip and no gas bubble in the filled electrode, the micropipette was put into the centrifuge (Spectrafuge 6c, Labnet). For the micropipette (outer diameter 1 mm, inner diameter 0.75 mm, and tip diameter 20–30 µm), centrifuging was used which, at 6500 rpm for 1–2 min, would have a good chance of no gallium leakage from the tip. However, these parameters depend on other factors too, e.g., the amount of the gallium filled into the micropipette, the profile of the micropipette, and the diameter of the micropipette tip. It may require a few runs to determine the speed and time duration for pipettes of different configuration. Then, the micropipette was trimmed to the desired diameter with a microforge.

*Measurement of the Electrical Resistance of Liquid Metal Electrodes*: The electrical resistance measurement setup is shown in Figure S2 (Supporting Information). The fabricated electrode tip was dipped into a pool of gallium. The pool of gallium as an electrode. An Ohmmeter was used to measure the resistance of the fabricated electrode by connecting it to the fabricated electrode and the gallium pool.

*Experimental Setup*: The schematic of the system setup is shown in Figure 4. The system consists of an inverted microscope (Axio Observer D1m, Zeiss), a motorized micromanipulator (Eppendorf, InjectMan NI 2), the multifunctional pipette, an oscilloscope, and a function generator. The pipette was mounted on the motorized micromanipulator to allow 3D positioning. The three liquid metal electrodes used in the demonstration experiments were connected to the function generator. A 35 mm Petri dish with cell was placed on the microscope stage. The pipette was carefully lowered to the bottom of the Petri dish with the joystick control of the micromanipulator. Then, AC signals were applied to the electrodes for trapping and rotating cells near the pipette tip with DEP and ROT mechanisms.

*Cell Preparation*: HeLa and PANC‐1 cells were cultured in Dulbecco's modified Eagle's medium (DMEM) supplemented with 10% fetal bovine serum (FBS) (Thermo Scientific) and 1% penicillin–streptomycin (Mediatech, Inc.) in a humidified atmosphere of 5% CO_2_ at 37 °C. Ramos B cells were cultured in RPMI 1640 (Mediatech, Inc) supplemented with 10% FBS and 1% penicillin–streptomycin in a humidified atmosphere of 5% CO_2_ at 37 °C. Before manipulation, cells were suspended in DMEM and mixed with the isotonic buffer (8.5% sucrose, 0.3% dextrose) whose electrical conductivity was adjusted to 0.028 S m^−1^ with KCl.

*DEP Force Calibration*: The experimental setup was used to trap cell with different applied voltage. The cell suspended in isotonic buffer is added on the Petri dish and place on the microscope stage. The pipette was mounted with a 60° to the substrate. Then, the pipette tip was placed near (≈5 µm) an isolated single cell. An AC signal was applied to the electrodes. A camera (FL3‐U3‐88S2C‐C, FLIR) was used to capture the trapping process at 21 frames per second. Then, the trapping process was repeated at different voltages. The cell location at each frame was extracted using ImageJ. The extracted data were used to compute the trap force.

*Cell Viability Test*: The experimental setup discussed in Figure 4 was UV sterilized for 30 min before conducting the experiment. A single microwell polydimethylsiloxane chip which had 0.5 mm diameter and 0.5 mm deep (Figure S1, Supporting Information) was used to prevent the cell being flushed away during medium change. Cells suspended in the isotonic buffer were dispensed near the microwell with a pipette. The pipette was mounted vertically to the microwell. Then, the pipette was used to transfer 30 cells into the microwell. The typical processing time was 30 min. The surplus cells were flushed away by replacing the isotonic buffer with a culture medium. The microwell was then put into the incubator. The cells were stained with calcein AM to count the number of live cells at 12 h or 3 d, depending on the viability test requirement. Cell viability was calculated as the ratio of the number of calcein AM positive cells at 12 h after DEP manipulation to the number of cells right after DEP manipulation. The proliferation rate was defined as the ratio of the total number of cells 3 d after DEP manipulation to the number of cells right after the DEP manipulation. The fluorescence images were captured for analysis by ImageJ.

## Conflict of Interest

The authors declare no conflict of interest.

## Supporting information

Supplementary

Supplementary

Supplementary

Supplementary
