# Supplementary material for: Liquid Metal‐Based Multifunctional Micropipette for 4D Single Cell Manipulation
Source: Adv Sci (Weinh). 2018 May 3;5(7):1700711. doi: 10.1002/advs.201700711 (PMC6051373; doi:10.1002/advs.201700711)
Supplement: Supplementary file 1 — Supplementary [file ADVS-5-1700711-s002.pdf]

Copyright WILEY-VCH Verlag GmbH & Co. KGaA, 69469 Weinheim, Germany, 2018.

ADVANCED SCIENCE
Open Access

Supporting Information

for *Adv. Sci.*, DOI: 10.1002/advs.201700711

Liquid Metal-Based Multifunctional Micropipette for 4D Single Cell Manipulation

*Yu Ting Chow, Tianxing Man, Giovanny F. Acosta-Vélez, Xiongfeng Zhu, Ximiao Wen, Pei-Shan Chung, Tingyi “Leo” Liu, Benjamin M. Wu, and Pei-Yu Chiou**

Copyright WILEY-VCH Verlag GmbH & Co. KGaA, 69469 Weinheim, Germany, 2016.

Supporting Information

Liquid Metal-Based Multifunctional Micropipette for Four-Dimensional Single Cell Manipulation

*Yu Ting Chow,[‡] Tianxing Man,[‡] Giovanny Acosta, Xiongfeng Zhu, Ximiao Wen, Tingyi "Leo" Liu, Benjamin M. Wu and Pei-Yu Chiou**

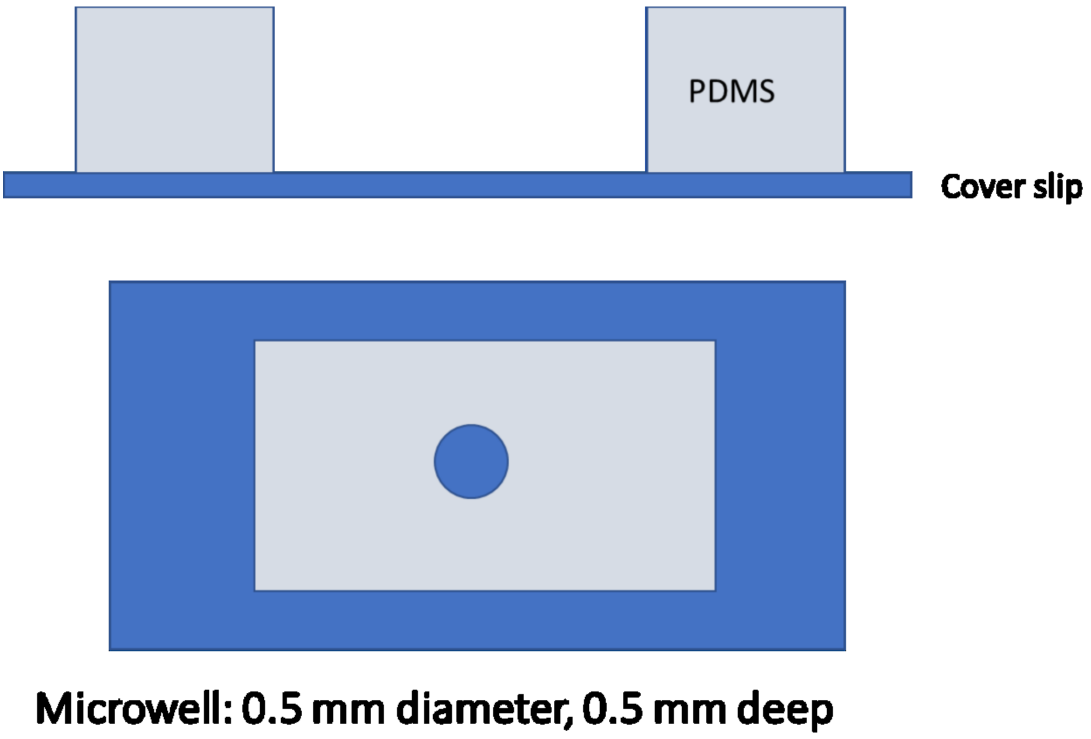

Figure S1. The schematic design of the polydimethylsiloxane (PDMS) microwell. The microwell has a 0.5mm diameter and is 0.5mm deep. This PDMS microwell is plasma bonded on a glass cover slip.

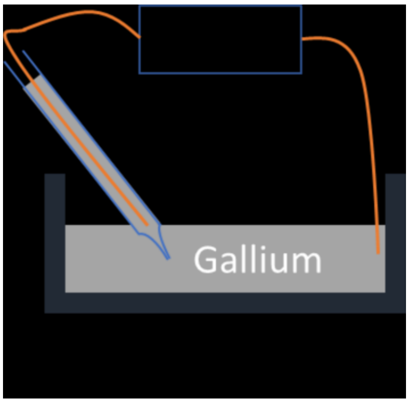

Figure S2. The experimental setup for measuring the electrical resistance of the fabricated electrode. The electrode tip is dipped into a pool of gallium. The pool of gallium works as an electrode. An ohm meter is used to measure the resistance of the fabricated electrode by connecting it to the fabricated electrode and the gallium pool.

Movie S1. A live Hela cell experiences positive DEP and trapped at the pipette tip. The live Hela cell was stained with calcein AM for identification.

Movie S2. A dead Hela cell experiences negative DEP and not trapped by the pipette. The dead Hela cell was stained with propidium iodide to identifty it.

Movie S3. Electrorotation of a PANC-1 cell. An 400 kHz, 4 V_{pp} AC signal is applied to the electrodes.
